# Extracellular Nucleophosmin Is Increased in Psoriasis and Correlates With the Determinants of Cardiovascular Diseases

**DOI:** 10.3389/fcvm.2022.867813

**Published:** 2022-04-28

**Authors:** Marco D'Agostino, Sara Beji, Sara Sileno, Daniela Lulli, Laura Mercurio, Stefania Madonna, Corrado Cirielli, Sabatino Pallotta, Cristina Albanesi, Maurizio C. Capogrossi, Daniele Avitabile, Guido Melillo, Alessandra Magenta

**Affiliations:** ^1^Experimental Immunology Laboratory, Istituto Dermopatico dell'Immacolata (IDI-IRCCS), Rome, Italy; ^2^National Research Council of Italy (CNR), Institute of Translational Pharmacology (IFT), Rome, Italy; ^3^Unit of Vascular Surgery, Istituto Dermopatico dell'Immacolata (IDI-IRCCS), Rome, Italy; ^4^Division of Dermatology, Istituto Dermopatico dell'Immacolata (IDI-IRCCS), Rome, Italy; ^5^Division of Cardiology, Johns Hopkins Bayview Medical Center, Johns Hopkins University, Baltimore, MD, United States; ^6^Laboratory of Cardiovascular Science, National Institute on Aging (NIA), National Institutes of Health (NIH), Baltimore, MD, United States; ^7^Idi Farmaceutici S.r.l., Pomezia, Italy; ^8^Unit of Cardiology, Istituto Dermopatico dell'Immacolata (IDI-IRCCS), Rome, Italy

**Keywords:** alarmin, inflammation, microRNAs, psoriasis, atherosclerosis, cardiovascular diseases

## Abstract

We previously showed that genotoxic stress induced an active extracellular release of nucleophosmin (NPM) in human cardiac mesenchymal progenitor cells, and that serum deprivation provokes NPM secretion from human endothelial cells, eliciting inflammation *via* nuclear factor kappa B (NF-kB) transcriptional activation. In this study, we wanted to determine whether NPM was similarly modulated in the skin and plasma of psoriatic patients (Pso). We found that NPM was induced in 6 skin biopsies compared to 6 normal skin biopsies and was markedly increased in lesional (LS) vs. non-lesional skin (NLS) biopsies. Moreover, NPM was also increased at the transcriptional levels in LS vs. NLS. Both the innate stimuli, such as lipopolysaccharides and Poly inositol–cytosine and adaptive stimuli, that is, cytokine mix, were able to induce the extracellular release of NPM in immortalized keratinocytes and human skin fibroblasts in the absence of cytotoxicity. Interestingly, NPM interacts with Toll-like receptor (TLR)4 in these cells and activates an NF-kB-dependent inflammatory pathway upregulating interleukin IL-6 and COX-2 gene expression. Finally, circulating NPM was increased in the plasma of 29 Pso compared to 29 healthy controls, and positively correlates with psoriasis area severity index (PASI) and with determinants of cardiovascular diseases (CVDs), such as pulse wave velocity, systolic pressure, and left ventricular mass. Furthermore, NPM positively correlates with miR-200c circulating levels, which we previously showed to increase in Pso and correlate with CVD progression. Our data show that circulating miR-200c is physically associated with extracellular NPM, which most probably is responsible for its extracellular release and protection upon cytokine mix *via* a TLR4-mechanism. In conclusion, NPM is increased in psoriasis both in the skin and plasma and might be considered a novel biologic target to counteract chronic inflammation associated with CVD risk.

## Introduction

Nucleophosmin (NPM) is a ubiquitous multifunctional nucleolar phosphoprotein that shuttles between the nucleolus and cytoplasm displaying chaperone-like activities (1, 2).

The intracellular roles of NPM include a wide range of metabolic processes such as ribosome biogenesis, proliferation, cell cycle control, migration, DNA repair, non-programmed cell death, stress sensor (3, 4).

It also regulates the stability and activity of transcription factors including p53 and nuclear factor kappa B (NF-κB) (5, 6).

In particular, NPM is directly associated with the DNA-binding domain of NF-kB p65 subunit, enhancing its DNA-binding activity. Moreover, NPM is crucial for inflammatory gene expression induced by tumor necrosis factor alpha (TNF-α) and lipopolysaccharides (LPS) in fibroblasts and macrophages (6).

In psoriasis, the over-proliferation of basal layer keratinocytes (KCs) is coupled with differentiation and cornification defects in the upper layers, leading to plaque formation (7). Interestingly, the NPM level is enhanced in proliferating KCs in psoriatic lesions as compared with healthy and uninvolved skin (8).

Nucleophosmin has been described also in the extracellular milieu, it has been shown that LPS treatment induced extracellular NPM release by human macrophages, and extracellular NPM, in turn, activates inflammatory pathways, suggesting an alarmin-like function, that is, a molecule released from damaged or diseased tissue that stimulates an immunomodulatory response (9).

We recently showed that following double-strand break formation, caused either by Doxorubicin (Dox) or ultraviolet radiations (UV), human cardiac mesenchymal progenitor cells (hCmPCs) actively release NPM in the extracellular space, in the absence of apoptosis, by an unconventional autophagic-dependent mechanism that requires Toll-like receptor (TLR)4 (10). The extracellular NPM induced a decrease in cell proliferation and it physically binds to TLR4, activating NF-kB-dependent inflammatory pathways (10). Moreover, NPM levels are increased in the plasma of Dox-treated mice (10).

We also showed that upon serum starvation, NPM is rapidly secreted by human umbilical vein endothelial cells (HUVEC) and acts as a pro-inflammatory and angiogenic molecule both *in vitro* and *in vivo* (11).

microRNAs (miRNAs) are short non-coding RNA molecules that modulate the translational efficiency and/or stability of target messenger RNAs (mRNA) (12).

miRNAs are not only intracellular but are also released in the extracellular space in body fluids (e.g., in serum, plasma, urine, saliva, and milk). They are protected by RNAse, since they are contained either in small membranous vesicles or packaged within HDL-cholesterol or linked to RNA-binding proteins (13).

Interestingly, NPM displays a nucleic acid–binding domain (14), it has been shown that a fraction of miRNAs, actively released in the extracellular space by cancer cells upon serum deprivation, are bound to NPM, and hence are protected from degradation (15).

We previously showed that circulating miR-200c is upregulated in chronic inflammatory diseases, such as atherosclerosis (16) and psoriasis (17).

In psoriasis, we found a positive correlation between miR-200c levels and disease severity and some determinants of cardiovascular diseases (CVD) (17).

In this study, we wanted to determine whether NPM is secreted in the extracellular space also by skin cells of healthy and psoriatic patients following activation with inflammatory stimuli. Moreover, we sought to determine whether extracellular NPM (eNPM) exerts inflammatory functions, as we found in hCmPCs, and whether eNPM correlates with disease severity and determinants of cardiovascular disease (CVD).

Finally, we investigated whether circulating miR-200c is, at least in part, protected by eNPM, and thus eNPM and miR-200c levels positively correlated in chronic inflammatory diseases.

## Materials and Methods

### Subject Selection and Classification

Patients affected by chronic plaque psoriasis (Pso, *N* = 29) and controls (Ctrl, *N* = 29) were prospectively enrolled from 2014 to 2016 at the Istituto Dermopatico dell'Immacolata (IDI)-IRCCS, Rome, Italy. All patients, afferent to the Integrated Research Centre for Psoriasis of IDI-IRCCS, had severe psoriasis defined by the Psoriasis Area and Severity Index (PASI) >10 and at least one of the following conditions: previous history of at least two systemic treatments for psoriasis, psoriasis onset before 40 years of age, and psoriasis disease duration of more than 10 years.

Exclusion criteria were diabetes, history of cerebrovascular events, myocardial infarction and/or myocardial revascularization, and psoriatic arthritis. Additional exclusion criteria were positivity for hepatitis B (HBsAg, anti-HBc), hepatitis C (anti-HCV), human immunodeficiency virus (anti-HIV 1 and 2); presence of a cancer or a hematologic disease; pregnancy or breastfeeding; neuropsychiatric diseases interfering with the patient's collaboration to the study; radiotherapy; chemotherapy; or systemic treatment with corticosteroids or immunosuppressive agents. Anthropometric data, including gender, weight, height, and body mass index (BMI), were measured for all subjects.

Clinical features were also recorded, including disease duration, blood pressure, medications, and smoking habits, and are reported in Supplementary Tables 1–3 and were previously described (17).

### Plasma and Skin Samples

Plasma samples were collected from peripheral blood of 29 Pso and 29 Ctrl subjects following the standard laboratory procedures. In all subjects, blood samples were obtained from the antecubital brachial vein after an overnight fast of at least 8 h and used to measure total cholesterol, low-density lipoprotein (LDL) cholesterol, high-density lipoprotein (HDL) cholesterol, and blood glucose levels. Venous blood samples (10 ml) were collected in EDTA-containing tubes. Blood was then centrifuged (1,200 × g for 10 min at 4°C), and supernatants were collected and centrifuged (12,000 × g for 10 min at 4°C). Plasma samples were stored at −80° C and thawed on ice before use.

About 6 mm punch biopsies were taken from LS deriving from the plaques at sites of peripheral lesions. Skin biopsy from NLS, normal-appearing (uninvolved) skin distant from the plaques, was also taken from the same Pso patients (*N* = 6). Patients had severe chronic plaque psoriasis (PASI ≥ 10). Pso patients were not undergoing topical treatments for at least 2 weeks before the study. Skin biopsies were immediately frozen in nitrogen liquid at −180° C.

### Transthoracic Echocardiography

A single experienced cardiologist performed a comprehensive two-dimensional (2D) transthoracic echocardiographic examination in all patients, using a commercially available device (Vivid 7, GE Vingmed Ultrasound AS, Horten, Norway). Data acquisition was performed with a 2.5–3.5 MHz transducer in the parasternal and apical views (standard parasternal long-axis and short-axis, apical, four-chamber, and two-chamber views). Pulsed-wave and continuous-wave Doppler analysis of flow velocities, as well as Color Doppler and Tissue Doppler analysis, were routinely used to obtain echocardiographic parameters according to the American Society of Echocardiography standards (18). The LV mass index was calculated as the ratio between the LV mass and body surface area (BSA).

### Blood Pressure Measurement

Systolic peripheral (brachial) pressure measurement and pulse wave velocity (PWV) calculation were performed by using a validated, commercially available system (SphygmoCor XCEL, AtCor Medical, Sydney, Australia), as previously described (19–21). Wave reflection analysis consisted of central (aortic) pressure assessment, together with the calculation of two indirect indexes of aortic stiffness: augmentation index and augmentation pressure. Carotid–femoral PWV, a direct index of aortic stiffness (21–23), was calculated as the distance traveled by the pulse wave divided by the time difference between the feet of carotid and femoral arterial waveforms. A PWV cutoff value >8.0 m/s was used as a marker of increased arterial stiffness in asymptomatic patients (23, 24).

### Cell Culture

Human keratinocytes (KCs) were established from biopsies obtained from NLS of psoriatic patients and skin of healthy subjects (HS) undergoing plastic and vascular surgery.

KCs were cultured on a feeder layer of lethally irradiated 3T3–J2 cells, according to the method described by Rheinwald and Green (25). Briefly, 2 cm^2^ skin biopsies were minced and trypsinized (0.05% trypsin/0.01% EDTA) at 37°C for 3 h. Afterward, cells were collected every 30 min, plated (2 x 10^6^/75-cm^2^ flask) on lethally irradiated 3T3–J2 cells, and cultured in 5% CO_2_ in KC growth medium (KGM): Dulbecco–Vogt Eagle's and Ham's F12 media (3:1 mixture) containing 10% FCS, insulin (5 μg/ml), transferrin (5 μg /ml), adenine (0.18 mM), hydrocortisone (0.4 μg/ml), cholera toxin (0.1 nM), triiodothyronine (20 pM), EGF (10 ng/ml), and penicillin/streptomycin (50 IU/ml). Confluent primary cultures were then trypsinized and cells were plated in secondary cultures at a density of 4 x 10^3^ to 1.3 x 10^4^ cells/cm^2^. Staining with a pan-cytokeratin antibody (AE13, Santa Cruz) was used to verify that the isolated cells were KCs.

Second- or third-passage cultured KCs were used in all experiments, with cells cultured in the serum-free medium KGM (Clonetics, San Diego, CA, USA) for at least 3–5 days (about 70% confluence) before performing treatments. Cultures of human fibroblasts (HFs) were established from NLS of psoriatic patients and HS undergoing plastic surgery. Briefly, full-thickness skin biopsies were cleaned with surgical scissors to remove most of the subcutaneous tissue and the remaining skin was finely minced into small pieces. These pieces were placed in a 75-cm^2^ culture flask and cultured in a fibroblast growth medium (DMEM containing 10% FBS, 50 IU−50 μg/ml penicillin–streptomycin and 4 mM glutamine) to obtain human fibroblast cultures (HF). The subconfluent primary HF cultures were amplified two times in a 1:6–1:7 split ratio. Prolyl-4-hydroxylase (C2, Santa Cruz) staining was used to be sure that isolated cells were HFs.

Subconfluent third-passage HF cultures were used for further molecular experiments. Immortalized keratinocytes (HaCaT) cells were grown in Dulbecco's modified Eagle's medium (DMEM) supplemented with 10% fetal bovine serum (FBS; Euroclone).

### Drug Treatments and Inflammatory Stimuli

Cells were treated with the following drugs administered in a serum-free medium: recombinant NPM (rNPM) was purchased from Abcam (Ab114194) and used 0.25 μg/ml; NPM inhibitor {N1,N2-bis[(3-imino-6-methyl-3H-indol-2-yl)methyl]-N1,N2-bis((6-methyl-1H-benzo[d] imidazol-2-yl)methyl)ethane-1,2-diamine hydrate, NSC348884 Sigma–Aldrich} was dissolved in DMSO and used 200μM, TLR4 inhibitor (TLR4i) is a small peptide [1-methylethyl 2-(acetylamino)-2-deoxy-α-D-glucopyranoside 3,4,6-triacetate, C34 5373/10 Tocris Bioscience] was dissolved in water and used 100 μM; RNAse was used 2 μg/ml. Stimulations with recombinant human IFN-γ (200 U/mL), TNF-α (50 ng/mL), IL-22 (50 ng/mL), IL-17A (50 ng/mL; R&D Systems, Minneapolis, MN, USA), LPS 100 μg/ml and 0.5 μg of Poly inositol–cytosine (I:C) both high molecular weight (HMW) and low molecular weight (LMW), were performed in a serum-free medium.

### Immunofluorescence

Subconfluent HaCaT and HFs grown on coverslips were treated with rNPM. Cells were fixed 30 min with 4% paraformaldehyde in PBS and permeabilized for 5 min with 0.1% Triton X-100. The following primary antibodies diluted in 1% BSA were incubated for 1 h at 37°C in a humid chamber: α-p65rel NF-kB (1:200; F6 Santa Cruz). Mouse IgG isotype control (Santa Cruz) was used at the same concentration of primary antibody. After PBS washes, secondary antibodies goat α-rabbit IgG-Texas Red (1:200 in PBS; Jackson Immunoresearch Laboratories, West Grove, PA, USA) were added and incubated for 1 h at 37°C in a humid chamber. Nuclei were stained with DAPI (1:10,000 in PBS; Sigma). Images were acquired with the ApoTome System (Zeiss) connected with an Axiovert200 inverted microscope (Zeiss); image analysis was performed with ZEN software (Zeiss).

### *In situ* Proximity Ligation Assay

The *in situ* proximity ligation assay (PLA) (26) was performed with the DuoLink *in situ* detection kit (Sigma) to detect and quantify the interaction of NPM with TLR4. Cells were grown on slides in 35 mm plates, fixed with 4% paraformaldehyde solution for 10 min, rinsed with PBS, and incubated for 1 h in the blocking buffer. α-NPM1 (ab15440; 1:400) or α-TLR4 (sc-293072; 1:100), α-rabbit PLUS/MINUS, and α-mouse PLUS/MINUS PLA probes were incubated for 1 h at 37°C and then washed. Hybridization, ligation, and amplification steps were performed for 30 min and polymerase reaction for 120 min at 37°C. Nuclei were counter-stained with DAPI (1:10,000 in PBS; Sigma). Images were captured with the ApoTome System (Zeiss) as described above. HFs and HaCaT cells were seeded on square glass coverslips. Subsequently, coverslips were removed and processed for PLA.

The PLA was quantified by counting the red dots and diving it for the number of nuclei per field, identified by DAPI staining by two independent observers, blinded to the status of the specimens. For each experiment, ~80–100 nuclei per treatment were accomplished. Six different primary HFs derived from healthy subjects and 5 different HaCaT experiments were performed.

### Immunohistochemistry

Paraffin-embedded sections were obtained from biopsies of healthy or psoriatic skin, including LS and NLS areas of evolving plaques. Five-micrometer sections were dewaxed and rehydrated. After quenching endogenous peroxidase, achieving antigen retrieval and blocking non-specific binding sites, sections were incubated with the anti-human NPM rabbit polyclonal antibody (ab15440; 1:200). Rabbit IgG isotype control (Santa Cruz) was used at the same concentration of primary antibody. Secondary biotinylated polyclonal Abs and staining kits were obtained from Vector Laboratories. Immunoreactivity was visualized with peroxidase reaction using 3-amino-9-ethylcarbazole (AEC, Vector Laboratories, Burlingame, CA) in H_2_O_2_ and specimen counterstained with hematoxylin. As a negative control, primary Abs was omitted. Stained sections were analyzed with the AxioCam digital camera coupled to the Axioplan 2 microscope (Carl Zeiss AG, Oberkochen, Germany). NPM staining intensity was evaluated by quantitative analysis (Image J color deconvolution) in three adjacent fields of each section by two independent observers, blinded to the status of the specimens. The percentage of IHC positivity areas was shown in dot plots graphs.

### ELISA Assay

KCs, HFs, and HaCaT cells were seeded on 6-well plates. After 8 h treatment with a mix of cytokines, supernatants were collected and frozen at −80°C. A 100 μl of supernatants or 5 μl of human plasma was used for NPM–ELISA (DBA Italia, SEC664Hu). NPM levels were measured by absorbance at 450 nm by EnSight microplate reader (Perkin Elmer).

### Cytotoxicity Assay

HaCaT and HFs were either serum starved or treated with 100 μg/ml LPS, or 0.5 μg of Poly I:C (HMW e LMW), or with a MIX of cytokines (IL-17A, IL-22, TNFα, and IFNγ) for 8 h and 24 h.

CellTox Green Cytotoxicity Assay (Promega) was used to test cytotoxicity. The fluorescence was measured at 500 nm Ex/530 nm Em at EnSight microplate reader (Perkin Elmer).

### Western Blot Analysis

Cells were lysed in a buffer containing 100 mM Tris (pH 6.8), 20% glycerol, and 4% sodium dodecyl sulfate (SDS). Protein concentration was determined by BCA protein assay kit (Pierce). Then, dithiothreitol 200 mM was added and lysates were boiled for 5 min. Proteins were separated by SDS–polyacrylamide gel electrophoresis (SDS–PAGE) and transferred to nitrocellulose membrane by standard procedures. The membranes were blocked with 5% non-fat dry milk powder in 0.05% Tween 20 phosphate-buffered saline (PBS-T) for 1 h. Immunodetection was performed by incubating the membranes with different primary antibodies overnight at 4°C. After four washes with PBS-T, the membranes were incubated with secondary antibody conjugated with horseradish peroxidase for 1 h. After washes, blots were developed with Amersham-ECL-Plus and exposed to ChemiDoc (Bio-Rad, Hercules, CA, USA). Protein levels were evaluated by densitometric analysis using Image Lab Software (Bio-Rad). Protein expression was normalized for α-tubulin protein levels. The following primary antibodies were used to detect the proteins of interest: anti-NPM1 (ab15440; Abcam) and anti-α-tubulin (Ab-1) (Oncogene Research Products).

### RNA Extraction and Analysis

RNA extraction was performed from 200 μl of plasma samples. miRNAs were isolated using Total RNA Purification Plus Kit (Norgen Biotek, Thorold, ON, Canada) according to the manufacturer's protocol, as previously described (27). As an internal control, 10 ftmoles of cel-miR-39a was spiked into each plasma sample after lysis. miRNA levels were analyzed using the TaqMan quantitative real-time PCR (qRT-PCR) and quantified with the QuantStudio5 real-time PCR (Thermo Fisher Scientific, Massachusetts, United States). Primers for miR-200c and cel-miR-39a and the reagents for reverse transcriptase and qPCR reactions were all obtained from Applied Biosystems. miRNA expression levels in each sample were normalized to cel-miR-39a. Relative expression in the fold was calculated using the comparative Ct method (2–ΔΔCt) (28).

Total RNA, from skin samples and HaCaT, HFs, and KCs, was extracted using, respectively, an RNeasy tissue lipid kit (Qiagen, Valencia, CA, USA) and QIAzol (Qiagen). cDNA was generated by the SuperScript First-Strand Synthesis System (Invitrogen), and real-time PCR was performed with the SYBR-GREEN RT-qPCR method (Qiagen) using QuantStudio5 Realtime-PCR. mRNA expression was normalized to 18S rRNA. Relative expression was calculated using the comparative Ct method (2–ΔΔCt).

The following primers were used for RT-qPCR:

COX-2

Forward 5′CTTCACGCATCAGTTTTTCAAG-3′

Reverse 5′TCACCGTAAATATGATTTAAGTCCAC-3′

IL-6

Forward 5′GATGAGTACAAAAGTCCTGATCCA-3′

Reverse 5′CTGCAGCCACTGGTTCTGT-3′

NPM

Forward 5′GAAAAAGGTGGTTCTCTTCC-3′

Reverse 5′TTTCCTCCACTGCCAGAGAT-3′

18S

Forward 5′CGAGCCGCCTGGATACC-3′

Reverse 5′CATGGCCTCAGTTCCGAAAA-3′

### Statistical Analysis

All data are expressed as means ± standard error (SEM) from at least 3 independent experiments. Because of the novelty of the study, whose primary objectives are mainly descriptive and exploratory, the minimum sample size has not been predetermined. Each variable was checked for normality distribution by the D'Agostino and Pearson omnibus normality test. The difference between the two groups was compared either by the two-tailed Mann–Whitney or Wilcoxon rank test for non-parametric groups or by two-tailed Student's *t* test for parametric variables using GraphPad Prism software (Version 5.0). Correlation analyses were carried out using a Spearman's test. *p* < 0.05 was considered to be statistically significant.

## Results

### NPM Level Is Increased in Pso Skin

**Figure 1:**
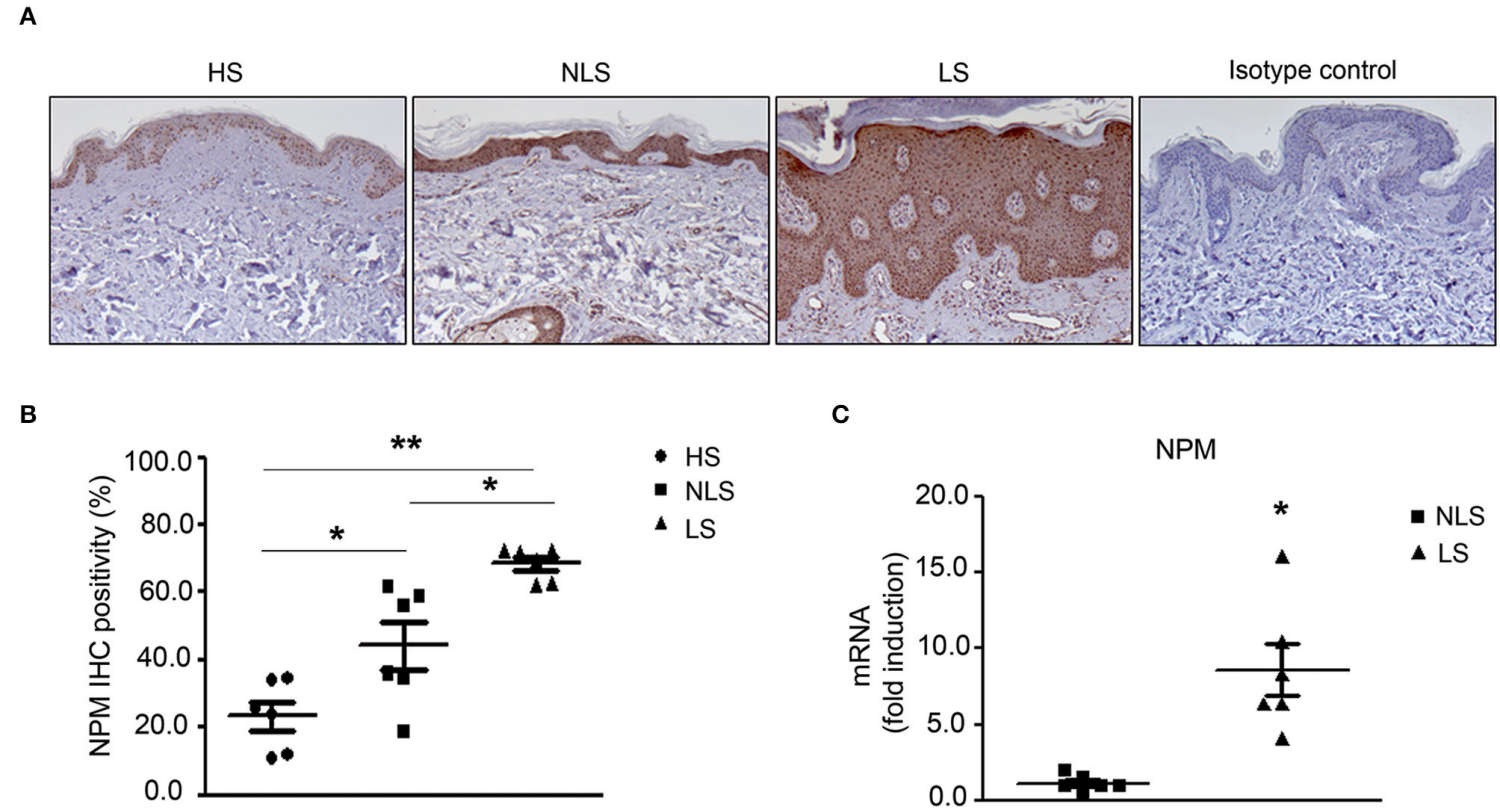
NPM protein level is increased in Pso skin. **(A)** Representative immunohistochemistry (IHC) of NPM. NPM protein level increased in human psoriatic patient paraffin-embedded biopsies of lesional skin (LS) compared to non-lesional skin (NLS) and biopsies of the skin of healthy individuals (HS). Rabbit IgG isotype control was used as a negative control. **(B)** IHC quantification of NPM of LS compared to NLS and HS. IHC was quantified by Image J color deconvolution of three adjacent fields for each section. Graph shows the IHC quantification as IHC positivity area expressed in percentage (*n* = 6; *p < 0.05, **p < 0.01). **(C)** NPM mRNA expression levels were increased in LS compared to NLS (*n* = 5; *p < 0.05).

Initially, we evaluated NPM levels in non-lesional (NLS) and lesional (LS) skin of psoriatic (Pso) patients by immunohistochemistry (IHC). We found that NPM level was higher in NLS compared to healthy skin (HS) (Figures 1A,B) and it was further increased in LS.

In agreement with these results, NPM mRNA expression was higher in LS compared to NLS biopsies (Figure 1C).

In conclusion, NPM mRNA and protein are highly increased in LS of Pso patients.

### Psoriasis and Inflammatory Stimuli Increased NPM Protein Levels in Both KCs and HFs

Afterward, we evaluated NPM protein levels in KCs and HFs extracted by Pso NLS and HS.

NPM was analyzed in KC and HF cultures in basal conditions and following their activation with a mix of cytokines (MIX) pathogenically associated with Pso condition (i.e., IL-17A, IL-22, TNF-α, and IFN-γ).

**Figure 2:**
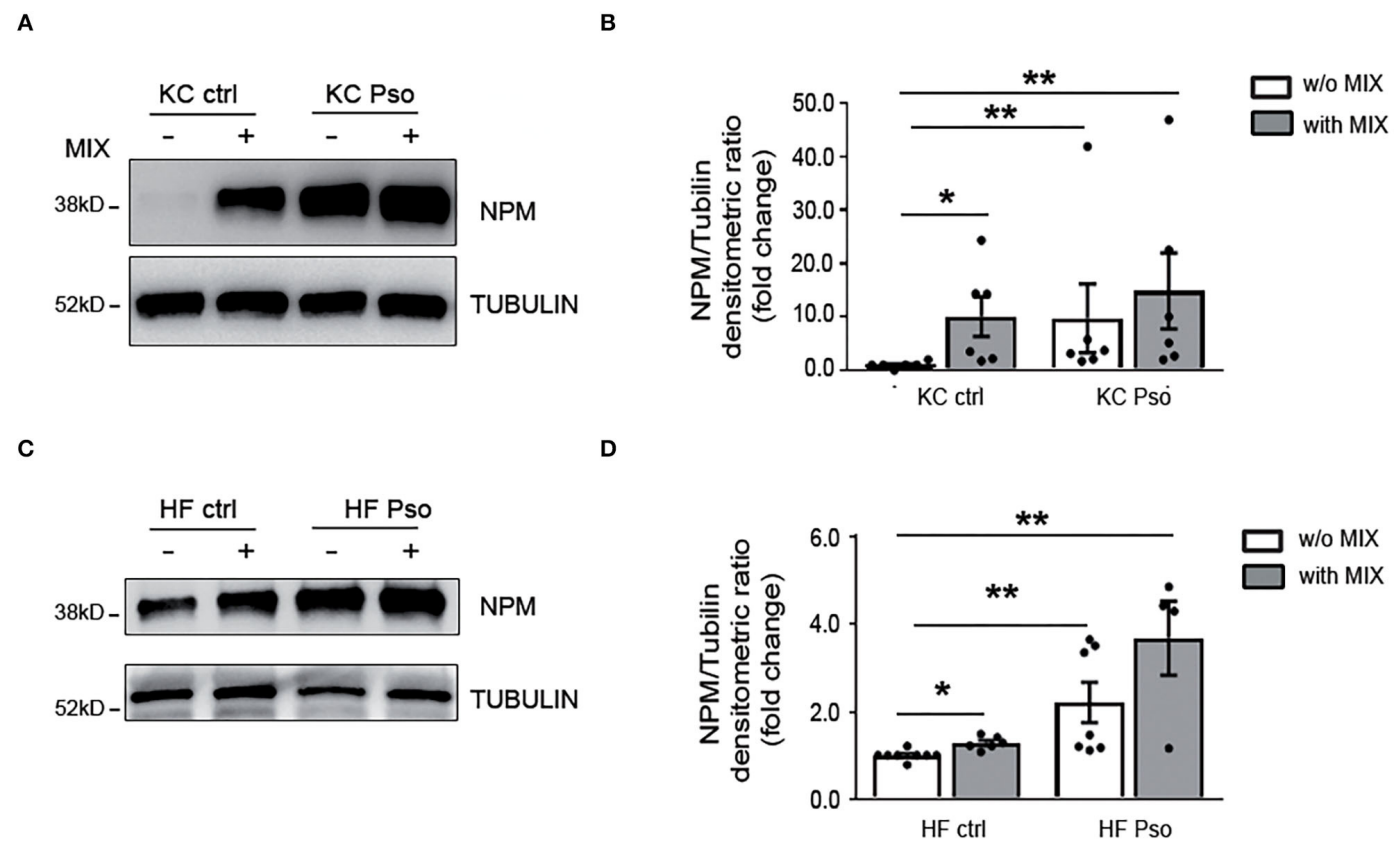
Psoriasis and inflammatory stimuli increased NPM protein levels in both KCs and HFs. Primary human keratinocytes (KCs) and human fibroblasts (HFs) were isolated from NLS of Pso and HS as control. KCs and HFs were treated for 8 h with a MIX of cytokines (IL-17A, IL-22, TNFα, and IFN-γ). Afterward, NPM protein levels were evaluated by Western blot analysis (WB). **(A)** Representative WB using NPM antibody showed that NPM protein expression was increased in KCs treated with the cytokine MIX and in KCs isolated from NLS of Pso compared to HS. **(B)** Densitometric analysis of NPM normalized by α-tubulin protein expression levels (*n* = 6; *p < 0.05, **p < 0.01). **(C)** NPM protein expression was increased in HFs isolated from NLS of Pso and in control KCs treated with the cytokine MIX compared to HS. **(D)** Densitometric analysis of NPM normalized by α-tubulin protein levels (*n* = 6; *p < 0.05, **p < 0.01).

We found that NPM protein was strongly increased in Pso KCs as compared to HS KCs and that the treatment with the cytokine MIX in HS KCs induced NPM protein at a similar level to that found in Pso KCs (Figures 2A,B). Pso KCs also showed NPM increased levels after MIX treatment, even if at a lower extent compared to HS KCs (Figures 2A,B).

In addition, Pso HFs showed higher amounts of NPM as compared to HF of healthy subjects. In HFs of healthy subjects, the cytokine MIX increased NPM levels (Figures 2C,D). In Pso HFs the MIX induced NPM protein level, but the increase did not reach statistical significance (Figures 2C,D).

We also found that cytokine MIX upregulated NPM mRNA in HaCaT cells (Supplementary Figure 1A), whereas in HFs and KCs NPM transcriptional upregulation by cytokine MIX did not reach statistical significance (Supplementary Figures 1B,C).

Therefore, NPM protein levels are increased in both KCs and HFs of Pso skin and the cytokine MIX mimics Pso condition.

### eNPM Is Rapidly Secreted by HaCaT, KCs, and HFs Upon Different Inflammatory Stimuli

**Figure 3:**
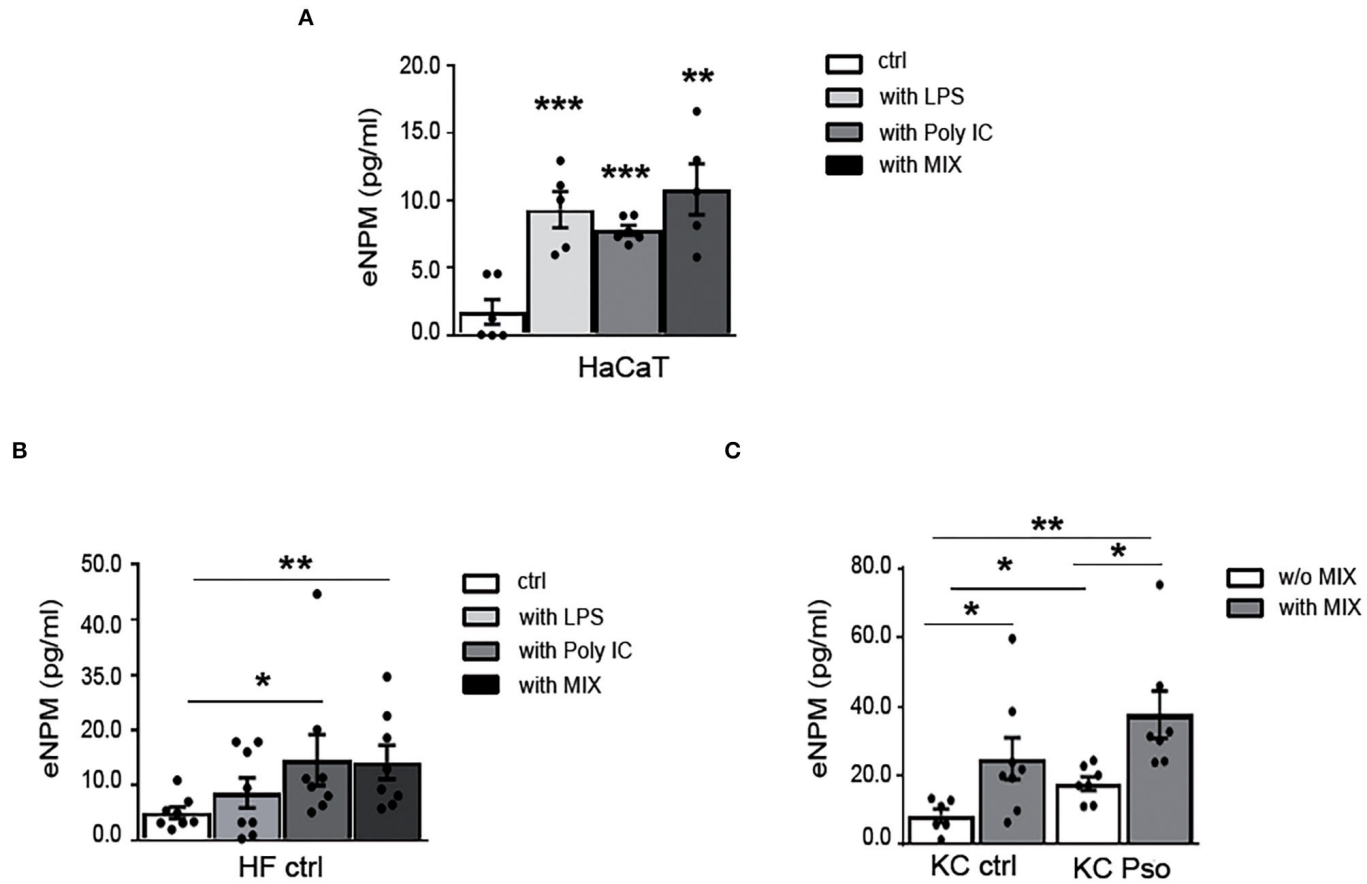
eNPM is rapidly secreted in the extracellular space by HaCaT, KCs, and HFs upon different inflammatory stimuli. **(A)** HaCaT were either serum starved for 8 h or treated with 100 μg/ml LPS, or 0.5 μg of Poly I:C (HMW e LMW), or with a MIX of cytokines (IL-17A, IL-22, TNFα, and IFNγ). Extracellular NPM levels (eNPM) were quantified by ELISA assay (*n* = 5; **p < 0.01, ***p < 0.001). All the treatments induced an increase eNPM compared to starvation. **(B)** HFs of healthy subjects were either serum starved for 8 h, or treated with 100 μg/ml LPS, or 0.5 μg of Poly I:C (HMW e LMW), or with a MIX of cytokines (IL-17A, IL-22, TNF-α, and IFN-γ). eNPM levels were quantified by ELISA assay (*n* = 8; *p < 0.05, **p < 0.01). Poly I:C and cytokine MIX increased eNPM significantly in the supernatants. **(C)** Healthy KCs and KCs of NLS of Pso were either serum starved or treated with a MIX of cytokines for 8 h. eNPM was quantified using a specific ELISA assay (*n* = 6; *p < 0.05, **p < 0.01). eNPM was increased in the supernatants of KCs isolated from NLS of Pso and in healthy KCs treated with the cytokine MIX compared to serum-starved healthy KCs supernatants. eNPM was also increased in the supernatants of KCs of Pso treated with cytokine MIX compared to serum-starved KCs of NLS of Pso supernatants.

We previously showed that NPM is released in the extracellular space in response to different stimuli that induce nucleolar stress in hCmPCs (10). Since NPM is highly expressed in proliferating cells of the basal layers of Pso skin, we investigated whether NPM was secreted in the supernatants by immortalized KCs (HaCaT) treated with either innate immunity mediators (LPS and Poly I:C) or a MIX of Pso-related cytokines (i.e., IL-17, TNF-α, IL-22, IFN-γ). A specific NPM–ELISA showed a significant increase of eNPM release in the supernatants of HaCaT at 8 h time point, with all the stimuli (Figure 3A). Subsequently, we wanted to verify whether this induction occurred in HFs of HS. All the stimuli induced eNPM but only the cells stimulated with either Poly I:C or with the MIX, reached statistical significance (Figure 3B). To exclude that NPM was passively released in the supernatants, we analyzed cytotoxicity at 8 and 24 h time points. We found that in both HaCaT and HFs at 8 h, all the stimuli did not induce cytotoxicity, whereas they all induced it at 24 h (Supplementary Figures 2A,B). In both cells, serum deprivation *per se* induced cytotoxicity at 24 h (Supplementary Figures 2A,B).

Moreover, we sought to determine whether eNPM was modulated in the supernatants of KCs isolated from HS or NLS of Pso biopsies, and whether the treatment with the cytokine MIX could influence its release. For this purpose, the KCs of HS and NLS of Pso were stimulated for 8 h with the MIX. We found that eNPM was higher in supernatants of NLS KCs compared to strains obtained from HS, and that in both cases the MIX further increases eNPM release (Figure 3C).

Taken together, these results indicated that eNPM can be actively released in response to different stimuli that mimic psoriasis in the absence of cytotoxicity, suggesting a functional role in this inflammatory condition.

### eNPM Binds to TLR4 and the Binding Increases Upon Cytokine Treatment in HaCaT and HFs

**Figure 4:**
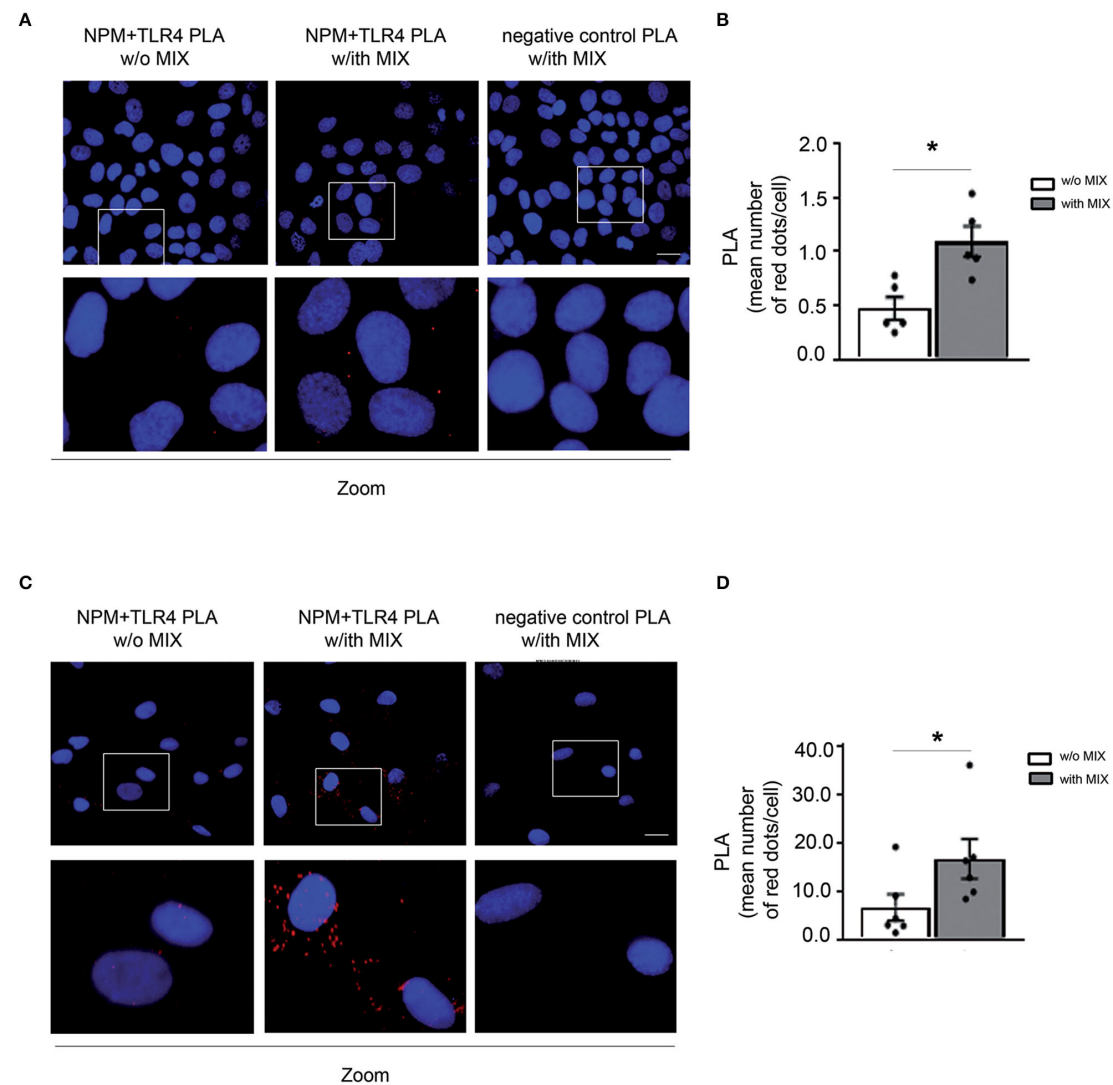
eNPM binds to TLR4 and the binding increases upon cytokines treatment in HaCaT and HFs. **(A)** Proximity ligation assay (PLA) of NPM and TLR4 interaction analysis in serum-starved HaCaT cells stimulated with a MIX of cytokines for 8 h (IL-17A, IL-22, TNF-α, and IFN-γ). Lower panels represent zoomed-in images of the original PLA images. Red dots indicate that even under physiological conditions, NPM interacts with TLR4. When a MIX of cytokines is administered, the rate of interaction increases. As a negative control, the IgG specific for each antibody (i.e., normal rabbit IgG used as the control of TLR4 antibody) and normal mouse IgG for NPM antibody (left panel) revealed the specificity of NPM and TLR4 interaction, since no red dots were present in the PLA. Scale bar 10 μm. **(B)** Bar graph of PLA interactions quantified by the mean number of red dots per cell (*n* = 5; *p < 0.05). **(C)** PLA of NPM and TLR4 interaction analysis in serum-starved HFs derived from healthy subjects and stimulated with a MIX of cytokines for 8 h. Lower panels represent zoomed-in images of the original PLA images. Scale bar 10 μm. **(D)** Bar graph of PLA interactions quantified by the mean number of red dots per cell (*n* = 6; *p < 0.05).

To investigate the possible mechanism of the autocrine/paracrine inflammatory effects of eNPM, we explored whether it interacts with Toll-like receptor 4 (TLR4), as we recently demonstrated in hCmPs (10). NPM and TLR4 interaction was confirmed *in situ* by proximity ligation assay (PLA) in HaCaT (Figures 4A,B) and HFs (Figures 4C,D) treated or not with the MIX for 8 h. In both cases, cell cultures showed an evident increase of NPM binding to TLR4 upon treatment, as revealed by the increasing number of detectable red dots (Figures 4A–D). The NPM–TLR4 interaction, upon MIX treatment was more appreciable in fibroblasts, where it was 3-fold upregulated (Figures 4C,D). These results indicated that in skin cells, eNPM binds to TLR4, suggesting a possible downstream signaling cascade activation.

### Recombinant NPM Promotes NF-κB Translocation to the Nucleus and Induces Inflammation in HaCaT *via* TLR4

**Figure 5:**
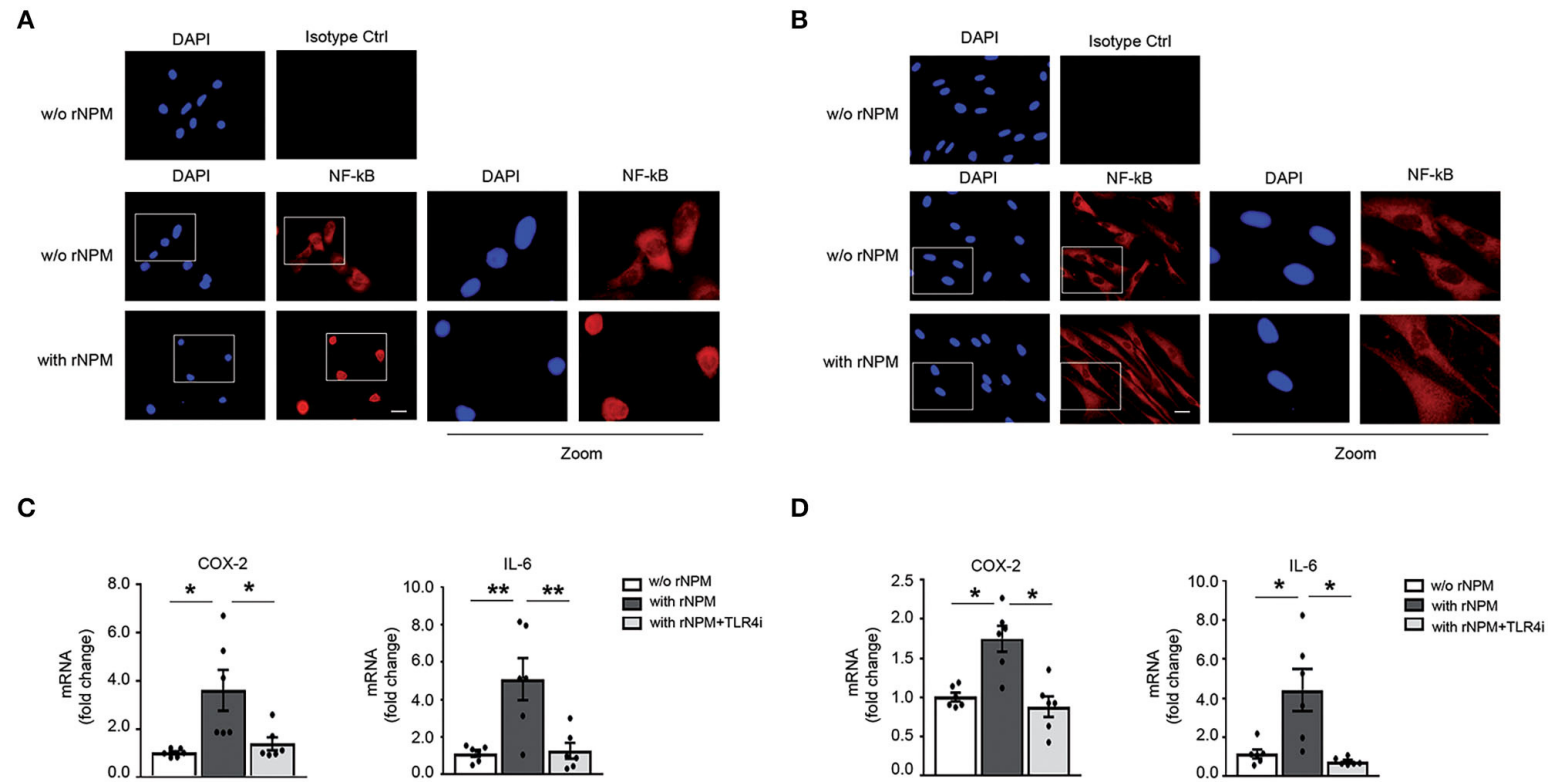
rNPM promotes NF-κB translocation to the nucleus and induces inflammation in HaCaT and HFs *via* TLR4. **(A)** Representative image of immunofluorescence of NF-kB (red) with an α-p65rel NF-kB antibody, nuclei were counter-stained with DAPI (blue). NFκB translocated in the nucleus in HaCaT treated for 8 h with rNPM (0.25 μg/ml) compared to untreated cells (w/o NPM). Mouse IgG isotype control was used as a negative control. Left panels represent zoomed-in images of the original images. Scale bar 5 μm. **(B)** Representative image of immunofluorescence of NF-kB (red) with an α-p65rel NF-kB antibody, nuclei were counter-stained with DAPI (blue). NF-κB translocated in the nucleus in HFs treated for 8 h with rNPM (0.25 μg/ml) compared to untreated cells (w/o NPM). Mouse IgG isotype control was used as a negative control. Left panels represent zoomed-in images of the original images. Scale bar 5 μm. **(C)** HaCaT were pre-treated with 100 μM TLR4i for 30 min and then treated or not for 8 h with rNPM (0.25 μg/ml) compared to untreated cells (w/o NPM). qRT-PCR of COX-2 and IL-6 was significantly reduced by TLR4i upon rNPM treatment (*n* = 6; *p < 0.05; **p < 0.01). **(D)** HFs were pre-treated with 100 μM TLR4i for 30 min and then treated or not for 8 h with rNPM (0.25 μg/ml) compared to untreated cells (w/o NPM). qRT-PCR of COX-2 and IL-6 was significantly reduced by TLR4i upon rNPM treatment (*n* = 6; *p < 0.05).

We showed in hCmPCs that recombinant NPM (rNPM) induced NF-kB nuclear translocation/activation (10), thus we wanted to determine whether this was also true in HaCaT and HFs. To this aim, an NF-κB immunofluorescence was performed using an anti-p65rel NF-kB antibody. We found that upon rNPM treatment, NF-κB was localized in the nucleus, whereas untreated cells showed a prevalent cytoplasm localization in both HaCaT (Figure 5A) and HFs (Figure 5B).

Moreover, to assess whether NF-κB translocation was followed by transcriptional activation of NF-κB target genes, we evaluated Cycloxygenase 2 (COX-2) and interleukin 6 (IL-6) mRNA expression in rNPM-treated vs. control in HaCaT and HFs. rNPM treatment induced both COX-2 and IL-6 mRNA expression, which was instead significantly decreased in the presence of a TLR4 inhibitor (TLR4i) a small molecule that inhibits TLR4 activity (29) (Figures 5C,D).

These findings indicated that eNPM/TLR4 interaction induces an NF-κB-dependent inflammatory signal transduction pathway in skin cells.

### TRL4-Based Mechanism Regulates NPM and miR-200c Secretion in Response to Cytokine Mix, and miR-200c and NPM Physically Interact

NPM displays a nucleic acid–binding domain and in response to serum starvation, was shown to be actively secreted by cancer cells and to be associated with and protect microRNAs from degradation in the extracellular space (15). Thus, we wanted to verify whether NPM interacts with and protects miR-200c, which we previously found to be increased both in LS and in plasma of Pso (17).

**Figure 6:**
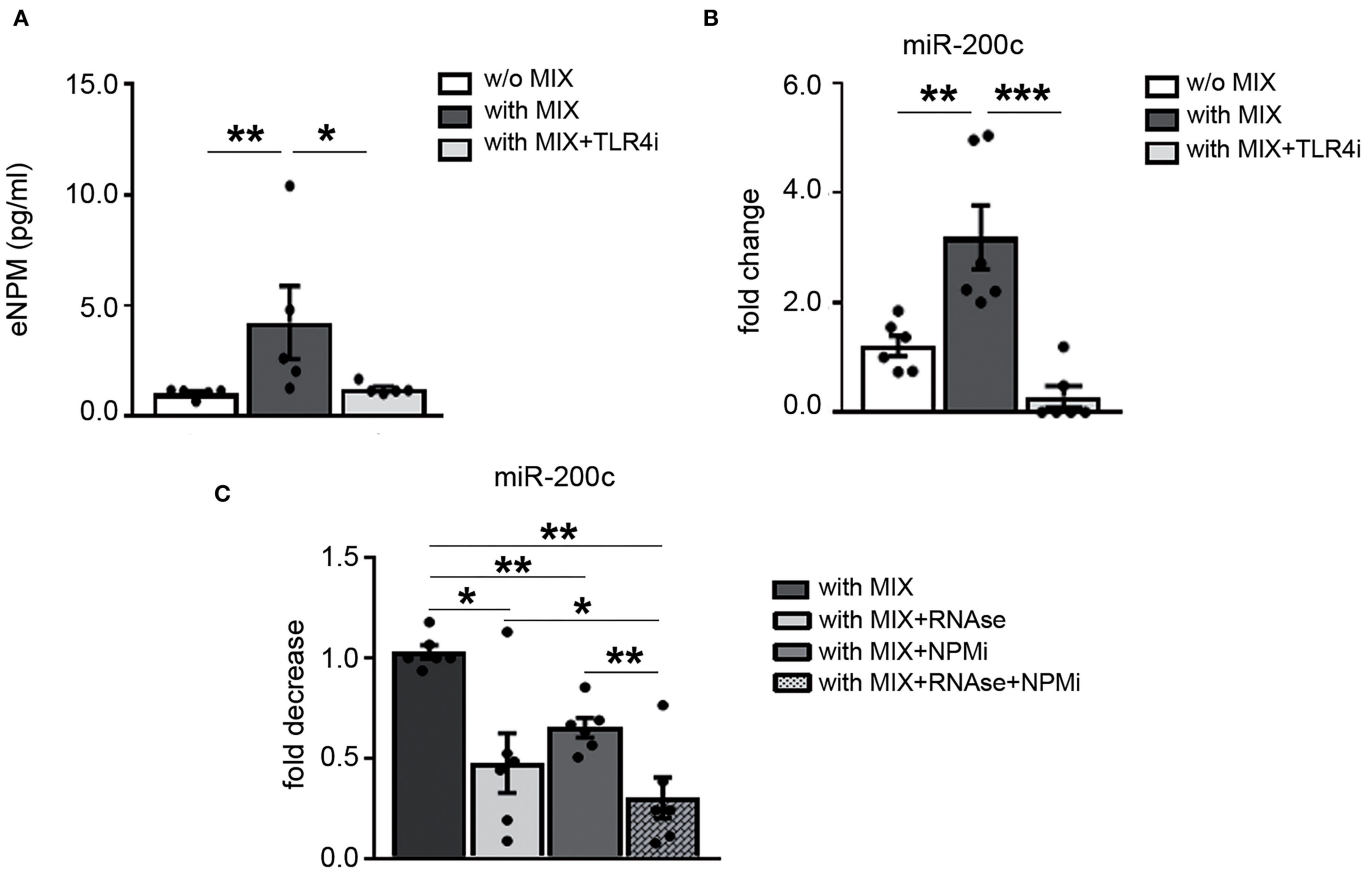
TRL4-based mechanism regulates NPM and miR-200c secretion in response to cytokine mix and miR-200c and NPM physically interact. HaCaT were pre-treated with 100 μM TLR4i for 30 min and then treated or not for 8 h with cytokine MIX (IL-17A, IL-22, TNF-α, and IFN-γ). **(A)** Media were collected and analyzed by ELISA assay for the presence of eNPM. eNPM increased levels by cytokine MIX were significantly decreased by TLR4i (*n* = 5; *p < 0.05; **p < 0.01). **(B)** qRT-PCR of miR-200c from the supernatant show that miR-200c increase elicited by cytokine MIX treatment is significantly reduced by TLR4i (*n* = 6; **p < 0.01; ***p < 0.001). **(C)** HaCaT were treated with cytokine MIX for 8 h then supernatants were collected and treated either with RNase or 200 μM NPM inhibitor (NPMi) or 2 μg/ml RNAse together with 200 μM NPMi for 15 min at 37°C (*n* = 6, ^*^p < 0.05; ^*^^*^p < 0.01).

We previously showed that NPM is released in the supernatants *via* a TLR4 mechanism in hCmPCs (10). To evaluate the possibility that NPM extracellular release by the MIX was based on a TLR4-dependent mechanism, HaCaT cells were exposed for 8 h to the MIX with or without TLR4i and eNPM levels were measured in the supernatants. We found that TLR4i significantly inhibited NPM secretion upon MIX exposure (Figure 6A).

Then, we verified if miR-200c was secreted by HaCaT upon MIX treatment, we choose these cells since miR-200c intracellular levels are higher than HFs. Our results showed that miR-200c levels were upregulated in the extracellular space of HaCaT by MIX treatment (Figure 6B). Subsequently, we evaluated whether TLR4 was involved also in miR-200c release. We found that miR-200c extracellular levels were reduced by TLR4i (Figure 6B).

Afterward, we asked whether NPM could interact and protect miR-200c in the supernatants of HaCaT stimulated with the MIX. To this aim, we used an NPM inhibitor (NSC348884) that inhibits NPM function disrupting its oligomer formation (30).

Our results indicated that miR-200c extracellular levels were decreased by NPM inhibitor (NPMi) treatment, demonstrating its binding to NPM. The treatment with RNAse alone also decreased miR-200c levels, but the concomitant treatment with NPMi further decreased miR-200c levels (Figure 6C).

Taken together, these results showed that TLR4 modulates NPM and miR-200c secretion by HaCaT cells and that miR-200c and NPM interact in the extracellular space, at least in part.

### eNPM Is Increased in Plasma of Psoriatic Patients and Correlates With miR-200c and Determinants of Cardiovascular Risk

The above-described data show that NPM is released by skin cells upon inflammatory stimuli. Since, we previously showed that NPM plays an important role as an alarmin in hCmPCs and HUVEC by regulating the immune response at a systemic level (10), we asked whether eNPM was increased in plasma of Pso.

**Figure 7:**
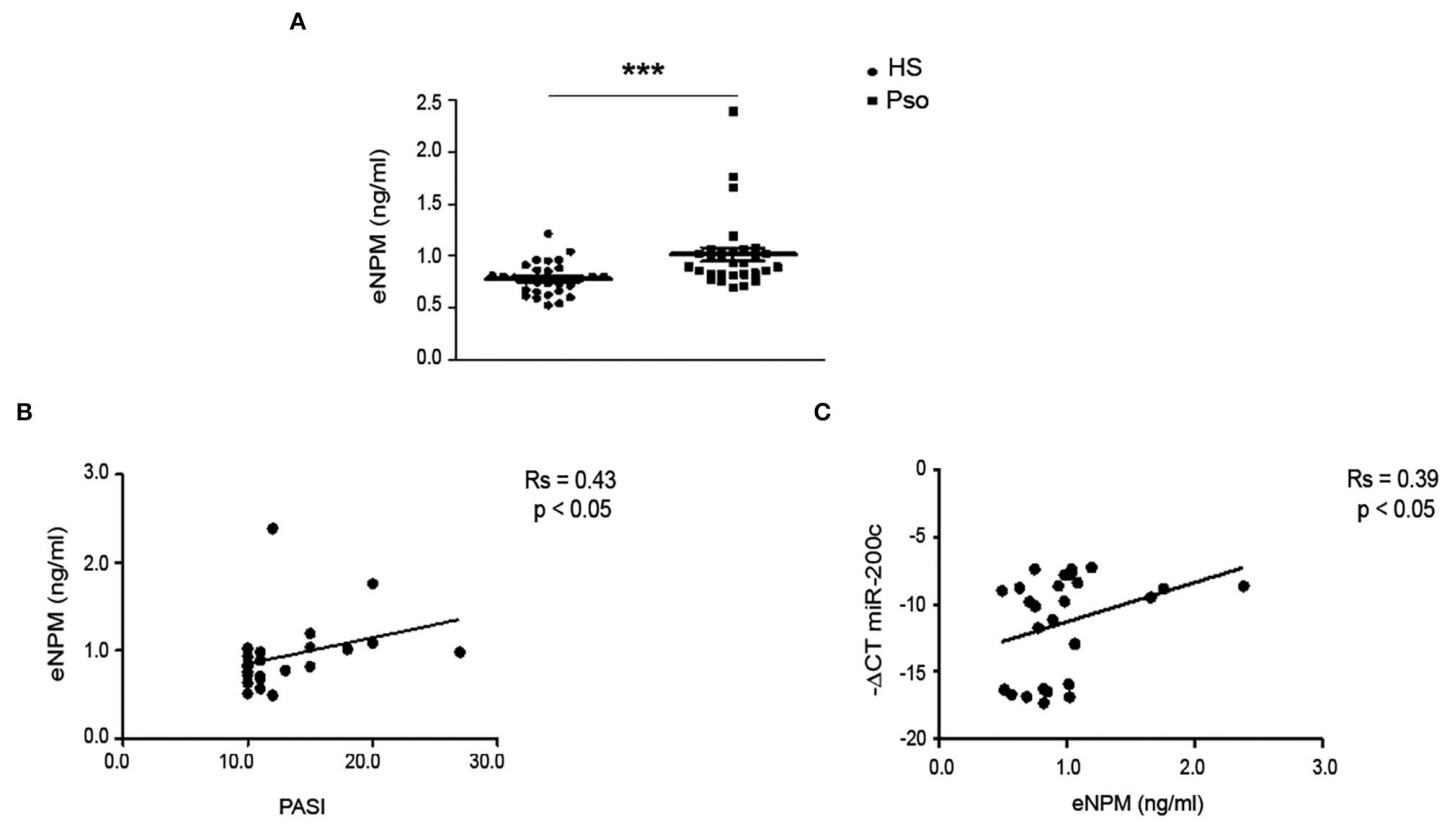
eNPM is increased in plasma of Pso patients and positively correlates with PASI and miR-200c. **(A)** Plasma of healthy control subjects (Ctrl) and psoriatic patients (Pso) was assayed for eNPM by ELISA **(A)** eNPM level was increased in Pso compared to Ctrl (Mann–Whitney test) (^***^p < 0.001). **(B)** Correlation analyses of eNPM levels with Psoriasis Area and Severity Index (PASI). **(C)** Correlation analyses of eNPM levels with miR-200c circulating levels (Spearman's correlation test).

We analyzed NPM expression levels in plasma of 29 Pso patients compared to 29 healthy controls (Ctrls), previously described (17, 31). We found that eNPM was significantly increased in Pso compared to Ctrl (Figure 7A). Interestingly, eNPM showed a positive correlation with the score of psoriasis severity PASI (Figure 7B). We then evaluated whether eNPM levels could correlate with circulating miR-200c, which we previously showed to be upregulated in the same plasma samples of Pso patients and to correlate with PASI and determinants of CVD (17). Interestingly, eNPM positively correlated with miR-200c expression levels (Figure 7C).

**Figure 8:**
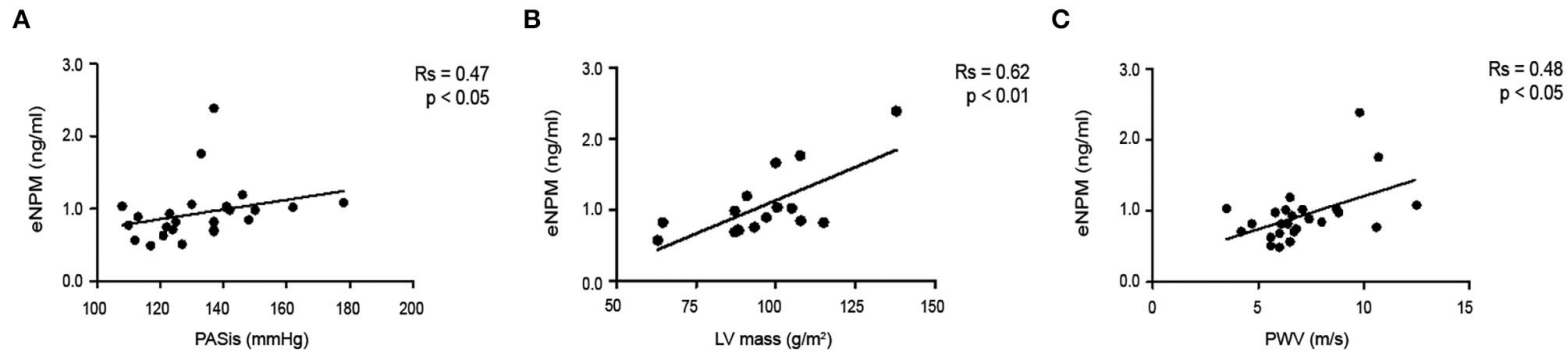
eNPM positively correlates with determinants of CV risk. Correlation of eNPM levels with: **(A)** Systolic Pressure (PASis); **(B)** left ventricular (LV) mass index; and **(C)** Pulse wave velocity (PWV) (Spearman's correlation test).

Subsequently, we tested whether circulating eNPM expression levels correlated with indexes of subclinical atherosclerosis. Our results showed that eNPM positively correlated with Systolic Pressure (PASis) (Figure 8A), left ventricular (LV) (Figure 8B) mass index, and pulse wave velocity (PWV) (Figure 8C).

Thus, eNPM levels are increased in Pso plasma and positively correlated with indexes of inflammation and cardiovascular disease progression.

## Discussion

In this study, we report, for the first time, the role of the nucleolar protein NPM as a possible biomarker of Pso severity and cardiovascular risk, confirming its role as an alarmin, involved in inflammatory pathways activation. These observations are in agreement with our previous studies reporting an NPM release in the extracellular milieu, as an early response to genotoxic stress response in hCmPCs (10) and serum deprivation in endothelial cells (11). Moreover, we previously described that in a mouse model of Dox-induced cardiotoxicity, circulating eNPM was increased in plasma, confirming NPM implication in CVD (10).

As previously reported (8), we confirmed that NPM protein is highly induced in hyperproliferating KCs in psoriatic skin lesion biopsies as compared to healthy and uninvolved skin, and we found that NPM is localized mainly in the nuclei but a cytoplasmic localization in KCs throughout the epidermis was also detectable. Moreover, NPM protein level is increased in both HFs and KCs derived from NLS of Pso patient biopsies compared to healthy subject skin. Moreover, we observed that NPM transcriptional levels were upregulated in LS vs. non-NLS.

Psoriasis is a systemic chronic inflammatory disease with an immunogenetic basis, which involves both adaptive and innate immune mechanisms, that can be triggered extrinsically or intrinsically (32).

The innate immune system is the first response developed to detect exogenous pathogens, such as viruses, bacteria, parasites, and toxins or to sense trauma and wounds. This first line of defense is activated to detect and destroy these agents and to initiate repair, but also to modulate the adaptive immune response that follows this first line. The adaptive immune system is a more specific response, leading to recovery from disease and protection against its return (33).

Interestingly, we found that adaptive immunity stimuli mimicked by Pso-related cytokine MIX, increased NPM levels in skin cells. Moreover, innate immunity mediators (LPS, Poly I:C) and cytokine MIX were both able to induce NPM extracellular release from skin cells.

These data suggest that NPM is released upon different inflammatory stimuli from cutaneous cells and that a chronic inflammatory state, such as psoriatic condition, can elicit a further increase of eNPM.

Toll-like receptors are transmembrane proteins that play a major role in both the innate and adaptive immune responses known to be integral processes in psoriasis (34). TLRs are expressed on different cells including macrophages, innate immune cells, as well as non-immune cells, such as fibroblast cells and epithelial cells. TLR4 expression increase has been described in peripheral blood mononuclear cells of psoriatic patients compared to healthy controls (35). Moreover, TLR4 polymorphisms have been associated with different autoimmune conditions, including chronic plaque psoriasis and psoriatic arthritis (34), suggesting a key role of this receptor in inflammatory pathways associated with psoriasis.

In addition, TLR4 has been shown to interact with alarmins (36) and our previous results demonstrated an interaction of eNPM with TLR4 in hCmPCs (10), therefore we tested TLR4 involvement in eNPM biological activities on skin cells.

We demonstrated NPM and TLR4 interaction by PLA, this technique was chosen for its sensitivity, and also because it allows us to localize the extracellular binding of NPM, since we did not permeabilize the cells. Serum deprivation *per se* induces NPM release in small quantities, but the interaction of NPM and TLR4 is further upregulated by cytokine MIX treatment. Interestingly, the binding was more evident in HFs compared to immortalized keratinocytes, although intracellular NPM seems to be higher in KCs upon cytokine treatment.

This could be due to a stronger responsivity of fibroblasts to inflammatory stimuli since TLR expression levels are higher in HFs compared to KCs (37).

As previously shown in hCmPCs (10), also in skin cells, eNPM is secreted *via* a TLR4-based mechanism and activates a TLR4 signaling cascade by inducing NF-kB translocation/activation in the nucleus. The transcriptional activation of NF-kB genes COX-2 and IL-6 was blunted by the inhibition of TLR4 with a small peptide that inhibits TLR4 activity. These results confirm an inflammatory role of eNPM acting as an alarmin *via* a TLR4/NF-kB mechanism also in skin cells.

Our previous studies demonstrated that oxidative stress upregulated miR-200c, and that miR-200c was responsible for reactive oxygen species increase, nitric oxide reduction, induction of apoptosis, and senescence, that finally causes endothelial dysfunction (38, 39). Moreover, we showed that miR-200c was upregulated in atherosclerotic carotid plaques and its expression was higher in unstable *vs* stable plaques. Interestingly, miR-200c levels correlated with inflammatory markers and with plaque instability biomarkers, and the circulating miR-200c levels were upregulated in the plasma of atherosclerotic patients compared to healthy subjects (16).

Psoriasis appears to be an independent risk factor for atherosclerotic CVD, this is probably due to the role that systemic inflammation plays in the induction of premature atherosclerosis and CVD in psoriatic patients (31, 40).

Different miRNAs have been linked to the pathogenesis of psoriasis and recently, we showed that miR-200c expression is upregulated in LS vs. NLS and plasma of Pso patients and is positively correlated with PASI and disease duration (17). We also found a positive correlation of miR-200c levels with determinants of CVD, that is, left ventricular (LV) mass index, relative wall thickness (RWT), and E/e′, a marker of diastolic dysfunction (17).

We found that Pso-related cytokines MIX induced extracellular release of miR-200c from immortalized KCs. We focused on these cells since KCs display a higher level of miR-200c compared to HFs. TLR4 was involved also in miR-200c release in the supernatants from KCs.

miRNAs in body fluids are protected by RNAse, because they are contained either in small membranous vesicles or packaged within HDL-cholesterol, or linked to RNA-binding proteins (13).

It has been shown that a fraction of miRNAs released by tumor cells upon serum deprivation are bound to NPM and hence, are protected from degradation (15). To assess the influence of NPM on miR-200c export and RNAse protection, we examined whether miR-200c extracellular levels were modulated by an NPM inhibitor that impairs its oligomeric formation (30). The NPM structural inhibitor decreased miR-200c levels in the immortalized KC supernatants upon cytokine MIX and the concomitant treatment with RNAse further decreases miR-200c levels, strongly indicating that at least a fraction of miR-200c is bound to NPM, and hence protected from degradation.

We cannot rule out that miR-200c is protected by degradation also by different ways besides NPM interaction, since the contemporary use of NPM inhibitor and RNAse treatment did not degrade miR-200c completely.

To further confirm these *in vitro* results, we verified whether eNPM was increased in the plasma of Pso patients compared to healthy controls. Indeed, eNPM levels were increased in Pso plasma and a positive correlation with miR-200c levels was found, therefore corroborating our hypothesis of an interaction between eNPM and miR-200c and of their concomitant release in the blood stream from inflamed skin tissues of Pso patients.

This is the first study that showed that NPM is secreted in the extracellular milieu in response to inflammatory stimuli and that NPM circulating levels are increased in chronic inflammatory diseases, such as Psoriasis.

Interestingly, eNPM positively correlated with PASI, an index of disease severity and inflammatory state. Moreover, eNPM in plasma correlated with some determinants of CVD risk, namely, with PASis, LV mass index, and PWV, which is a gold standard non-invasive measurement of arterial stiffness (21–23).

Taken together, the results of the present work suggest that the chronic inflammatory state and KC hyperproliferation that characterize psoriasis cause an intracellular increase of NPM expression in the skin. The latter is then actively released extracellularly upon innate and adaptive immune mediators *via* a TLR4-based mechanism. eNPM, in turn, exerts autocrine/paracrine biological functions responsible for the inflammatory pathway activation *via* direct binding to TLR4 and nuclear translocation/activation of NF-kB in both KCs and HFs.

Furthermore, miR-200c is released extracellularly *via* TLR4-dependent mechanisms and it physically interacts with NPM, which protects it from degradation, at least in part. It is possible to hypothesize that NPM and miR-200c are released together in the extracellular milieu from inflamed skin cells and they could act as inflammatory and immune response modulators in body fluids, affecting different tissue homeostasis, including the vasculature and cardiac tissues.

A study limitation is the small sample size of Pso patients used, and also the fact that echocardiographic analyses were not performed on all the patients. Notwithstanding this, we found positive correlations of eNPM levels with CVD risk parameters and with disease severity.

These patients were enrolled excluding the ones that already experienced major cardiovascular events, diabetes, or psoriatic arthritis, to find subclinical predictors of future CV events.

The increase of eNPM in plasma of Pso patients, suggests that it could be carefully considered in future studies with a larger sample size as a biomarker of disease severity and might be considered a useful therapeutic tool to be exploited for the reduction of the CVD risk and inflammation associated to psoriasis.

The feasibility of using an NPM inhibitor as a therapeutic tool is confirmed by the fact that two phases, two clinical trials, adopting two different NPM inhibitors for anti-cancer therapy in humans, showed to be safe, non-toxic, and well-tolerated and have been completed successfully [reviewed in (41)].

## Data Availability Statement

The raw data supporting the conclusions of this article will be made available by the authors, without undue reservation.

## Ethics Statement

The human plasma collection was approved by the Ethics Committee of IDI-IRCCS (Prot. no. 188/CE/2014 approved in 19/09/2014) and all patients provided written informed consent at enrollment. Skin biopsies were obtained from healthy subjects and Pso patients from lesional (LS) and non-lesional (NLS) areas of the same patient. Four different studies approved by the Ethics Committee of IDI-IRCCS were used (Prot. no. 34/CE/2016 approved in 14/10/2016, Prot. no. 47/CE/2016 approved in 18/11/2016, Prot. no. 44/CE/2019 approved in 04/07/2019, and Prot. no. 02/CE/2022 approved in 12/04/2018). All patients provided written informed consent at enrollment. The study was conducted following the Good Clinical Practice guidelines and according to the declaration of Helsinki. The patients/participants provided their written informed consent to participate in this study.

## Author Contributions

AM provided the study concept, designed the experiments, helped in RNA extraction, analyzed, interpreted the data, and wrote the article. MD'A conducted, performed experiments, analyzed, interpreted the data, wrote the paper, processed plasma, extracted RNA plasma, and performed the qRT-PCR. SB and SS collected clinical patient data, performed experiments, processed plasma, and extracted RNA from plasma. LM and SM performed experiments. DL helped in IHC experiments. SP, CC, and GM participated in plasma collection, patient enrolment, and performed surgical procedures. CA, DA, GM, and MC participated in analysis interpretation and article revision. All authors revised and approved the submitted manuscript.

## Funding

This study was partly supported by the Italian Ministry of Health RF02362708 grant to AM and MC; SG-12358253 grant to MD'A; by AFM Telethon 22522 grant to AM; POR A0375-2020-36572 to AM.

## Conflict of Interest

DA is employed by Idi Farmaceutici S.r.l. The remaining authors declare that the research was conducted in the absence of any commercial or financial relationships that could be construed as a potential conflict of interest.

## Publisher's Note

All claims expressed in this article are solely those of the authors and do not necessarily represent those of their affiliated organizations, or those of the publisher, the editors and the reviewers. Any product that may be evaluated in this article, or claim that may be made by its manufacturer, is not guaranteed or endorsed by the publisher.
