# Supplementary material for: Extracellular Nucleophosmin Is Increased in Psoriasis and Correlates With the Determinants of Cardiovascular Diseases
Source: Front Cardiovasc Med. 2022 Apr 28;9:867813. doi: 10.3389/fcvm.2022.867813 (PMC9095901; doi:10.3389/fcvm.2022.867813)
Supplement: Supplementary file 1 [file Table_1.docx]

Supplementary Table 1

| **Baseline clinical and laboratory parameters** | |  |  |
| --- | --- | --- | --- |
|  |  |  |  |
| **Characteristic** | **Control Healthy Subjects (n=29)** | **Psoriatic Subjects (n=29)** | **Comparison between**  **two groups**  **(P value)** |
| **Male Sex, N.** (%) | 19 (56) | 15 (52) | 0.42 |
| **Age** (years) | 49.00 ± 1.51 | 49.17 ± 2.50 | 0.95 |
| **BMI** (kg/m^2^) | 27.17 ± 0.88 | 28.62 ± 1.21 | 0.33 |
| **Waist circumference** (cm) | 102.90 ± 2.54 | 103.60 ± 2.79 | 0.85 |
| **Blood glucose** (mg/dl) | 96.24 ± 2.00 | 99.04 ± 6.76 | 0.68 |
| **Total cholesterol** (mgl/dL) | 193.30 ± 6.18 | 213.20 ± 6.89 | 0.04* |
| **LDL-cholesterol** (mgl/dL) | 120.40 ± 6.00 | 127.20 ± 8.90 | 0.53 |
| **HDL-cholesterol** (mgl/dL) | 52.44 ± 2.11 | 53.89 ± 2.46 | 0.66 |
| **Triacylglycerols** (mgl/dL) | 115.20 ± 11.82 | 107.60 ± 6.85 | 0.58 |
| **HsCRP** (mg/L) | 2.05 ± 0.76 | 8.97 ± 4.10 | 0.10 |
| **ESR** (mm/h) | 5.85 ± 1.52 | 13.27 ± 3.02 | 0.06 |
| **BSA** (m^2^) | 1.95 ± 0.04 | 1.91 ± 0.05 | 0.54 |
| **Diabetes mellitus, N.** (%) | 0 | 0 | 1 |
| **Ever Smoking, N.** (%) | 13(45) | 10(35) | 0.59 |
|  |  |  |  |
| Values are means +S.E.M. (*P<0.05, between groups). Comparisons between two groups were carried out by performing unpaired Student’s t-test for all variables with exception of the variable ‘Male sex’, 'Diabetes' and Ever Smoking for which a Fisher exact Test was performed. Abbreviations: BMI: Body Mass Index; LDL: low density lipoprotein; hs-CRP: high-sensitivity C-reactive protein; ESR: Erythrocyte sedimentation rate; BSA, body surface area | | | |
