# Supplementary material for: Extracellular Nucleophosmin Is Increased in Psoriasis and Correlates With the Determinants of Cardiovascular Diseases
Source: Front Cardiovasc Med. 2022 Apr 28;9:867813. doi: 10.3389/fcvm.2022.867813 (PMC9095901; doi:10.3389/fcvm.2022.867813)
Supplement: Supplementary file 2 [file Table_2.docx]

Supplementary Table 2

| **Echocardiographic parameters** | |  |  |
| --- | --- | --- | --- |
|  |  |  |  |
| **Characteristic** | **Control Healthy Subjects (n=23)** | **Psoriatic Subjects (n=17)** | **Comparison between**  **two groups (P value)** |
| **Male Sex, N.** (%) | 16 (70) | 10 (59) | 0.50 |
| **Age** (years) | 48.17+1.82 | 50.06 +3.31 | 0.60 |
| **LV mass index** (g/m^2)^ | 84.17 ± 5.23 | 102.50 ± 4.77 | 0.02* |
| **RWT** (cm) | 0.40 ± 0.01 | 0.48 ± 0.03 | 0.01** |
| **E/e'** | 7.41 ± 0.50 | 7.59 ± 0.44 | 0.80 |
|  |  |  |  |
| Values are means +S.E.M. (*P<0.05, **P<0.01 between groups). Comparisons between two groups were carried out by performing unpaired Student’s t-test for all variables with exception of the variable ‘Male sex’, for which a Fisher exact Test was performed. Abbreviations: LV: left ventricular; RWT: relative wall thickness. | | | |
|  |  |  |  |
|  |  |  |  |
