# Supplementary material for: Extracellular Nucleophosmin Is Increased in Psoriasis and Correlates With the Determinants of Cardiovascular Diseases
Source: Front Cardiovasc Med. 2022 Apr 28;9:867813. doi: 10.3389/fcvm.2022.867813 (PMC9095901; doi:10.3389/fcvm.2022.867813)
Supplement: Supplementary file 3 [file Table_3.docx]

Supplementary Table 3

| **Blood Pressure, Wave reflection analyses**  **and PWV** | | |  |  | |  |  |  |
| --- | --- | --- | --- | --- | --- | --- | --- | --- |
|  | |  |  |  | |  |  |  |
| **Characteristic** | | **Control Healthy**  **Subjects (n=29)** | | **Psoriatic**  **Subjects (n=29)** | | **Comparison between**  **two groups**  **(P value)** | | |
| **Male Sex, N.** (%) | | 19 (56) | 15 (52) | 0.42 | |  |  |  |
| **Age** (years) | | 49.00 ± 1.51 | 49.17 ± 2.50 | 0.95 | |  |  |  |
| **Central systolic pressure (**mmHg) | | 129.90 ± 2.60 | 133.8 ± 3.08 | 0.33 | |  |  |  |
| **Central diastolic pressure** (mmHg) | 80.86 ± 1.32 | | 82.14 ± 2.34 | 0.63 | |  |  |  |
| **Central pulse pressure** (mmHg) | | 37.97 ± 1.44 | 41.33 ± 1.91 | 0.16 | |  |  |  |
| **Augmentation pressure** (mmHg) | | 11.86 ± 1.11 | 14.15 ± 1.62 | 0.24 | |  |  |  |
| **Augmentation index** | | 29.72 ± 2.29 | 32.67 ± 2.63 | 0.40 | |  |  |  |
| **Systolic pressure** (mmHg) | | 119.80 ± 2.20 | 123.80 ± 2.76 | 0.25 | |  |  |  |
| **Diastolic pressure** (mmHg) | | 81.83 ± 1.38 | 82.56 ± 2.03 | 0.76 | |  |  |  |
| **PWV** (m/s) | | 7.23 ± 0.23 | 7.18 ± 0.40 | 0.91 | |  |  |  |
| Values are means +S.E.M.  Comparisons between two groups were carried out by performing unpaired Student’s t-test for all variables with exception of the variable ‘Male sex’, for which a Fisher exact Test was performed. Abbreviations: PWV: pulse wave velocity. | | | | | | |  |  |
|  |  |  |  |  |  |  |  |  |
|  |  |  |  |  |  |  |  |  |
