# Supplementary material for: Extracellular Nucleophosmin Is Increased in Psoriasis and Correlates With the Determinants of Cardiovascular Diseases
Source: Front Cardiovasc Med. 2022 Apr 28;9:867813. doi: 10.3389/fcvm.2022.867813 (PMC9095901; doi:10.3389/fcvm.2022.867813)
Supplement: Supplementary file 4 [file Data_Sheet_1.docx]

Supplementary figure legends

**Supplementary Figure 1. NPM mRNA upon cytokine MIX exposure. a)** HaCaT cells were treated with cytokine MIX (IL-17A, IL-22, TNF-α and IFN-γ) for 8h. NPM mRNA expression levels were upregulated in HaCaT upon cytokine MIX (n = 6; * P <0.05). **b**) HFs were treated with cytokine MIX (IL-17A, IL-22, TNF-α and IFN-γ) for 8h. NPM mRNA expression levels were upregulated although not significantly (n=6). **c**) KCs were treated with cytokine MIX (IL-17A, IL-22, TNF-α and IFN-γ) for 8h. NPM mRNA expression levels were upregulated although not significantly (n=6).

**Supplementary Figure 2. Cytotoxicity assay upon different inflammatory stimuli. a)** HaCaT were either serum starved or treated with 100μg/ml LPS, or 0.5 μg of Poly I:C (HMW e LMW), or with a MIX of cytokines (IL-17A, IL-22, TNFα and IFNγ) for 8h and 24h. Afterwards cytotoxicity assay was performed. Cytotoxicity assays showed no significant differences among the different treatments at 8h treatment. At 24h all the treatments induced cytotoxicity compered to 24h serum free. The 24h serum starvation *per se* induced cytotoxicity compared to 0h treatment (n=6; *p<0.05). **b)** HFs were either serum starved or treated with 100μg/ml LPS, or 0.5 μg of Poly I:C (HMW e LMW), or with a MIX of cytokines (IL-17A, IL-22, TNFα and IFNγ) for 8h and 24h. Afterwards cytotoxicity assay was performed. Cytotoxicity assays showed no significant differences among the different treatments at 8h treatment. At 24h all the treatments induced cytotoxicity compered to 24h serum free. The 24h serum starvation *per se* induced cytotoxicity compared to 0h treatment (n=6; *p<0.05)
