# Supplementary figures and images for: Extracellular Nucleophosmin Is Increased in Psoriasis and Correlates With the Determinants of Cardiovascular Diseases
Source: Front Cardiovasc Med. 2022 Apr 28;9:867813. doi: 10.3389/fcvm.2022.867813 (PMC9095901; doi:10.3389/fcvm.2022.867813)

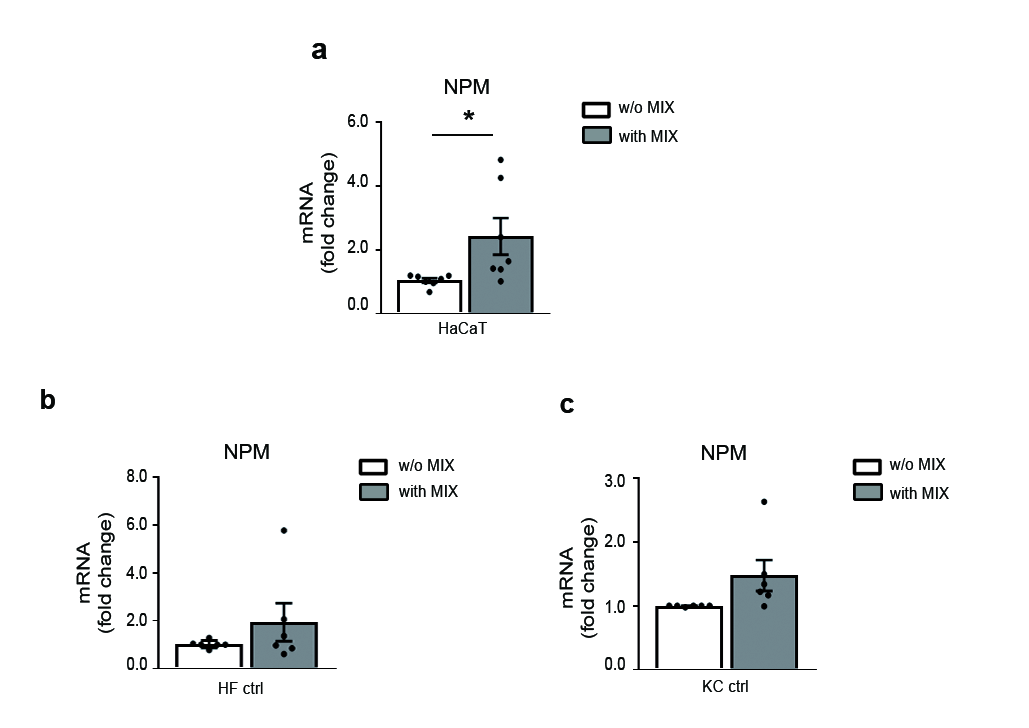

Supplement: Supplementary file 5 [file Image_1.TIF]

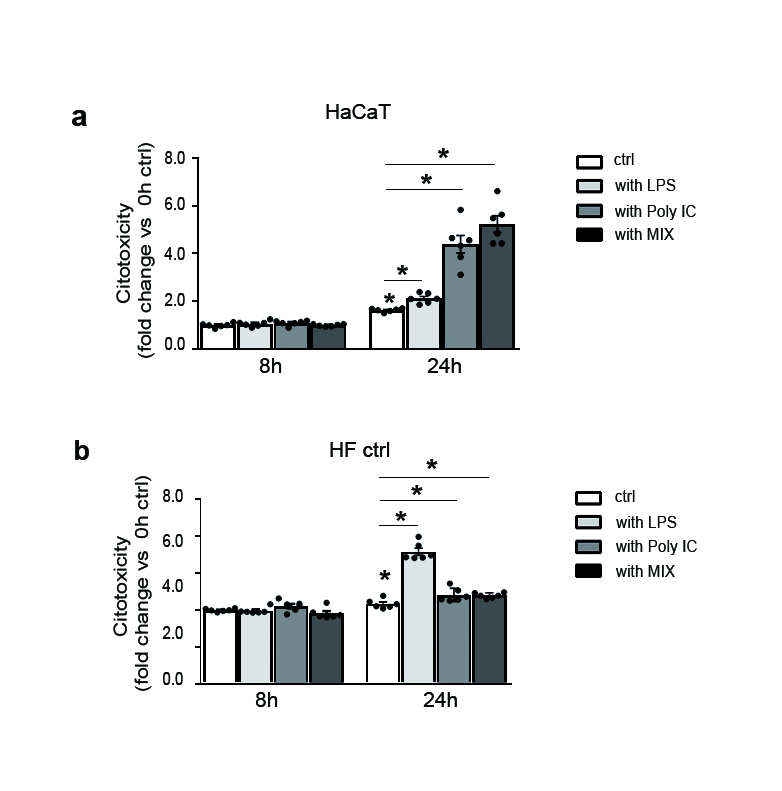

Supplement: Supplementary file 6 [file Image_2.TIF]
